# Prolonged Course of COVID-19-Associated Pneumonia in a B-Cell Depleted Patient After Rituximab

**DOI:** 10.3389/fonc.2020.01578

**Published:** 2020-09-02

**Authors:** Igor Kos, Benedikt Balensiefer, Sophie Roth, Manfred Ahlgrimm, Martina Sester, Tina Schmidt, Lorenz Thurner, Moritz Bewarder, Robert Bals, Frank Lammert, Stephan Stilgenbauer, Dominic Kaddu-Mulindwa

**Affiliations:** ^1^Department of Hematology, Oncology, Clinical Immunology, Rheumatology, Saarland University, Homburg, Germany; ^2^Department of Medical Microbiology and Hygiene, Institute of Medical Microbiology and Hygiene, Saarland University, Homburg, Germany; ^3^Department of Transplant and Infection Immunology, Saarland University, Homburg, Germany; ^4^Department of Pulmonology, Allergology and Critical Care Medicine, Saarland University, Homburg, Germany; ^5^Department of Gastroenterology, Hepatology, Endocrinology, Diabetology and Nutrition Medicine, Saarland University, Homburg, Germany

**Keywords:** COVID-19, SARS-CoV-2, B-NHL, T-cell activation, lymphoma

## Abstract

Patients with pre-existing comorbidities and immunosuppression are at greater risk for SARS-CoV-2 infection and severe manifestations of COVID-19. This also includes cancer patients, who are shown to have a poor prognosis after infection. Here, we describe the case of a 72-year old male patient with B-cell depletion after maintenance treatment with rituximab for non-Hodgkin-lymphoma who had a prolonged COVID-19 course and initial false negative test results. Our case highlights the diagnostic pitfalls in diagnosing COVID-19 in B-cell depleted patients and discuss the role of B-cell depletion in the course and treatment of COVID-19. Furthermore, we investigated peripheral blood monocytes and SARS-CoV-2 specific T cells in our patient. In conclusion, our case report can help physicians to avoid diagnostic pitfalls for COVID-19 in hemato-oncological patients under chemoimmunotherapy and tries to explain the role of B-cell depletion and SARS-CoV-2 specific T cells in this context.

## Introduction

It has been shown, that patients are at greatet risk for SARS-CoV-2 infection and severe course of COVID-19 if they have comorbidities and immunosuppression (1). We describe the case of a 72-year old male patient with a history of non-Hodgkin lymphoma and B-cell depletion due to treatment with rituximab, who had initial false negative test results and suffered from a prolonged COVID-19 course.

## Case Report

A 72-year-old man with history of nodal marginal zone lymphoma presented to our emergency department on April 2020 with 1 week of high fever and cough. He reported that a naso-pharyngeal swab (NPS) for SARS-CoV-2 testing had been performed by his primary care physician 1 week before admission and was negative. In 2017 he underwent chemoimmunotherapy with six cycles of bendamustine and rituximab, followed by rituximab maintenance therapy (2) from September 2017 to August 2019, achieving sustained complete remission. He had no other relevant comorbidities and no history of smoking. The patient was isolated in a single room until the negative result of the second NPS performed at admission was received. On day 2, he was transferred to the normal care unit of our oncology department.

Laboratory testing revealed elevated C-reactive protein (CRP) and lactic acid dehydrogenase levels, hypogammaglobulinemia, and anemia. Cellular immune status showed persistent B-cell depletion and CD4^+^ and CD8^+^ T-cell lymphopenia. Table 1 summarizes the laboratory findings over time. The serum levels of troponin, estimated glomerular filtration rate, and liver enzymes as well as an electrocardiogram were unremarkable. The patient had no hepato- or splenomegaly and the peripheral lymph node status (by physical examination) was also unremarkable. Arterial blood gas analysis showed a partial respiratory insufficiency with alkalosis (Table 1). Due to subpleural bilateral infiltrates in chest X-ray and considering the diagnosis of a community-acquired pneumonia, the patient was treated with intravenous ampicillin (2 g) and sulbactam (1 g) TID. Cytomegalovirus and Epstein-Barr virus reactivation were ruled out. Blood cultures showed no bacterial growth.

**Table 1:** Relevant laboratory findings.

**Laboratory values**	Day 2^*^	**Day 5**	**Day 17**
Hemoglobin level, g/L	11.7	12	9.7
Platelets level, cells/L	361	387	458
C-reactive protein level, mg/L	86, 6	114,4	85
Procalcitonin level, ng/ml	0.12	0.18	0.12
Lactic dehydrogenase level, U/L	377	311	315
Plasma IgG level, mg/dL	352		
Plasma IgM level, mg/dL	29		
Plasma IgA, level, mg/dL	76		
Glomerular filtration rate, mL/min	78.4	66.5	90.3
**Immune status**			
Leukocytes, 1/μl	5,390	6,700	5,600
B-lymphocytes, 1/μl	0	0	0
T-lymphocytes, 1/μl	931	975	1,054
CD4^+^ T cells, 1/μl	408	360	401
CD8^+^ T cells, 1/μl	489	587	644
NK-cells, 1/μl	29	17	27
HLA-DR+ lymphocytes	30%	29.1%	20%
CD4/CD8-ratio	0.82	0.61	0.62
**Monocytes**			
Total monocytes, 1/μl	n.d.	347	n.d.
HLA-DR+ monocytes, 1/μl (%)	n.d.	277 (80%)	n.d.
CD14^+^CD16^−^ monocytes, 1/μl (%)	n.d.	284 (82%)	n.d.
CD14^+^CD16^+^ monocytes 1/μl (%)	n.d.	45 (13%)	n.d.
CD14^−^CD16^+^ monocytes 1/μl (%)	n.d.	5.8 (1.6%)	n.d.
**Blood gas analysis**			
pH	7.506		
pCO_2_	31 mmHg		
pO_2_	63.9 mmHg		
Oxygen saturation	92%		
Oxygen supplementation	No		

* *Admission at oncology unit; pH, potential of hydrogen; pCO_2_, partial pressure of carbon dioxide; pO_2_, partial pressure of oxygen; mmHg, millimeter of mercury*.

**Figure 1:**
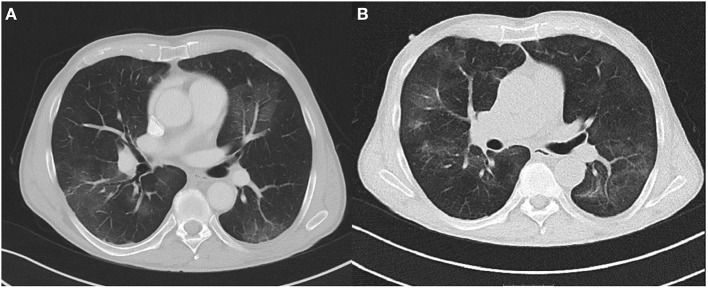
**(A)** Chest CT at day 5 showing bilateral peripheral ground-glass opacities. **(B)** Chest CT at day 17 showing opacities at different locations without significant reduction.

On day 5, the patient still presented with persistent fever. A chest CT was performed, which showed bilateral peripheral ground-glass opacities (Figure 1A). However, two additional RT-PCR tests from NPS for SARS-CoV-2 were negative. As expected, SARS-CoV-2 serological tests (IgA, IgG, and IgM) were negative, too. Due to the CT findings and still unidentified pathogen the patient underwent bronchoscopy with bronchoalveolar lavage (BAL). The results showed a positive RT-PCR for SARS-CoV-2 in BAL and bronchial aspirate. In addition, bacterial superinfection with *Haemophilus influenzae* and *Klebsiella oxytoca* was documented. The cytology was compatible with lymphocytic alveolitis with a CD4/CD8 ratio of 0.3.

**Figure 2:**
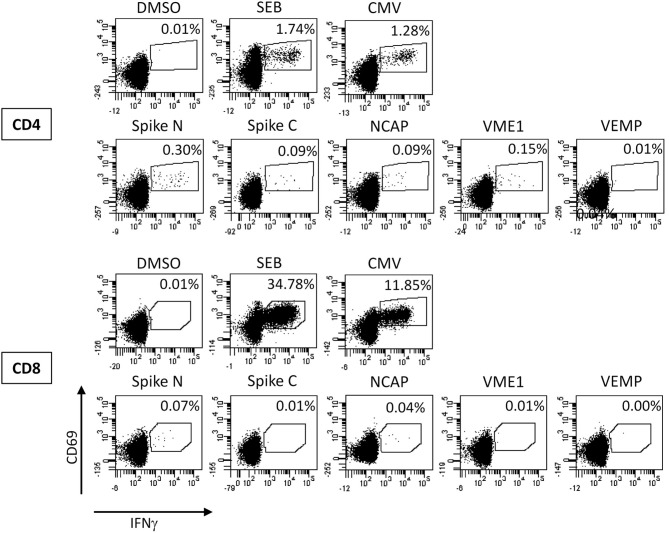
Analysis of SARS-CoV-2 specific T cells. Whole blood at day 19 was stimulated with overlapping peptide pools of the SARS-CoV-2 spike glycoprotein (divided in the N-terminal and C-terminal part, Spike N, and Spike C, respectively), the nucleocapsid protein (NCAP), the membrane protein (VME1), and the envelope small membrane protein (VEMP) for 6 h and antigen-specific CD4 and CD8 T cells were quantified based on the induction of CD69 and IFNγ. Stimulations with the solvent (DMSO), with *Staphylococcus aureus* Enterotoxin B (SEB), and with cytomegalovirus (CMV) antigens were carried out as negative and positive controls, respectively. Percentages in each dotplot refer to the percentages of specifically stimulated CD4 or CD8 T cells.

Based on these findings the diagnosis was changed to COVID-19 and the patient was transferred to our COVID-19 unit. At that time a subset analysis of the peripheral blood monocytes demonstrated an increased percentage of HLA-DR+ monocytes (80%) and moderately increased percentages of intermediate-type monocytes (CD14^+^CD16^+^, 13%; Table 1). In addition, the patient showed SARS-CoV-2 specific T cells primarily reactive toward peptide pools from the viral spike glycoprotein, nucleocapsid protein, and membrane protein (VME1, Figure 2). Specific cells with immediate effector function were mainly found among CD4 T cells (Figure 2). However, SARS-CoV-2 specific proliferation after 7 days was observed for both CD4 and CD8 T cells (7.8% of CD4 and 2.9% of CD8 T cells, data not shown).

In spite of antibiotic treatment with meropenem (1 g) and clarithromycin (500 mg) TID, follow-up chest CT at day 16 (Figure 1B) showed no significant improvement with multiple ground-glass opacities at different locations. The patient still had intermittent fever and elevated CRP levels. Therefore at day 17, we initiated treatment with high-dose intravenous immunoglobulin (Gamunex^®^, 25 g/d for 5 consecutive days). No adverse events were noted, and clinical and laboratory inflammation markers returned to normal. NPS remained negative for SARS-CoV-2, and the patient was discharged in improved clinical condition on day 24.

## Discussion

This report raises important aspects of COVID-19 in hemato-oncological patients. Firstly, standard diagnostics from NPS may be challenging in this patient group, and secondly immunochemotherapy and/or disease related immunocompromise may have influence on course and treatment of the disease.

RT-PCR from NPS is the established routine method for the diagnosis of SARS-CoV-2 (3, 4). Our patient underwent repeated tests carried out by well-trained staff according to the current guidelines. Hence, a false negative result due to insufficient technical sampling was highly unlikely. Recent studies have shown that the nasopharyngeal viral load is high during the first days of symptoms, decreasing thereafter. In some patients the virus can even be detected after resolution of symptoms (5, 6). Since viral load in the lower respiratory tract may remain higher for longer periods and decrease more slowly, this indicates that at the time of diagnosis, the virus was only present and therefore detectable in the patient's lungs (6, 7).

In general, chest CT provides fundamental information regarding the diagnosis of COVID-19. One study compared RT-PCR from NPS with chest CT, demonstrating similar sensitivity and specificity (3). Notably, the combination of both tests showed the best results (sensitivity of 94%) (3). In our patient, chest CT was also compatible with this diagnosis despite negative NPS. Considering that hemato-oncological patients have an increased infection-related mortality (62%) early chest CT and/or bronchoscopy with BAL can guide timely treatment of the patient and protection measures for health care staff (1). Of note in our case, neither exposed staff nor three family members of the patient tested positive for SARS-CoV-2 (data not shown).

To understand whether B-cell depletion has any influence on the disease course, it is important to characterize the role of these cells during COVID-19. Our patient had a considerably longer hospitalization time (24 days) compared to patients in recently published non-hematological cohorts (median of 10–12 days) (8, 9). Nevertheless, he only developed moderate symptoms and no severe complications such as coagulation disorders, stroke, or liver failure (8, 9). Similarly, one report of a rheumatoid arthritis patient treated with rituximab showed a considerably slower progression to severe disease compared to other cohorts (10). Two large cohorts of cancer patients with COVID-19 have identified risk factors associated with death. Those were higher age, male sex and the presence of comorbidities in both cohorts, whereas history of smoking, worse performance status and cancer activity despite of treatment was only seen in the cohort with American, Canadian and Spanish patients (11, 12). Despite of two underlying risk factors (age and sex) our patient still presented a positive outcome.

Preclinical research with SARS-CoV models has demonstrated that neutralizing antibodies against viral structures provide complete protection against infection (13, 14). Additionally, seroconversion can occur earlier than 7 days in COVID-19 patients (6, 7). Thus, the lack of an efficient antibody production (as shown by hypogammaglobulinemia) caused by B-cell depletion might explain the protracted disease course. B-cell depletion may on the other hand also provide an explanation for the moderate symptoms, as the lack of antibody-producing B cells may have prevented activation of the complement system. Moreover, non-neutralizing antibodies could worsen SARS-CoV-2 infection through antibody-dependent enhancement. Hence the lack of specific antibodies in our patient may have prevented antibody-mediated immunopathology. Our patient received high-dose intravenous immunoglobulins with good clinical recovery. The dose was determined based on immune modulation therapy for other diseases as well as individual COVID-19 cases (15).

Given the absence of specific antibodies, T cells may have contributed to the control of infection. Consistent with lymphopenia as one of the most prevalent features of COVID-19 (16), our patient had slightly reduced levels of CD4+ and CD8+ lymphocytes. Interestingly, the patient was able to mount a SARS-CoV-2 specific effector T-cell response with proliferative potential, which was dominated by CD4 T cells. This is remarkable, as bendamustine causes a very delayed T-lymphocyte reconstitution, which particularly affects CD4+ T cells (17). The BAL was PCR positive and showed an increased CD8+ T-cell count. Thus, although the antigen-specificity of BAL T cells was not determined, the dominance of SARS-CoV-2 specific CD4 T cells in blood may result from the fact that SARS-CoV-2 specific CD8 T cells may have homed to the lung as the site of infection. Apart from specific immunity, the patient showed a monocyte profile consistent with an activated innate immunity status.

## Conclusion

RT-PCR from NPS for SARS-CoV-2 might not be the diagnostic test of choice in hemato-oncological patients under chemoimmunotherapy, and other diagnostic strategies including earlier chest CT, RT-PCR from BAL, or determination of SARS-CoV-2 specific T cells need to be considered. The latter may be of particular interest as an alternative to seroconversion in patients treated with rituximab. The role of immunosuppression in the disease is not yet completely understood. B-cell depletion in our patient could have accounted for a longer but more moderate course of disease. In particular, the subgroup of B-cell depleted patients might benefit from treatment of COVID-19 with immunoglobulins. In addition, detection of SARS-CoV-2 specific T cells may emphasize a particular role of the cellular immune response for control of the infection that warrants further study.

## Data Availability Statement

The original contributions presented in the study are included in the article/supplementary material, further inquiries can be directed to the corresponding author/s.

## Ethics Statement

Ethical review and approval was not required for the study on human participants in accordance with the local legislation and institutional requirements. The patients/participants provided their written informed consent to participate in this study. Written informed consent was obtained from the individual(s) for the publication of any potentially identifiable images or data included in this article.

## Consent for Publication

The patient and his family have given consent for publication in an academic journal.

## Author Contributions

IK, BB, DK-M, SR, FL, LT, MA, MS, TS, SS, and MB contributed to collection, review, and/or analysis of the data. IK and BB wrote the manuscript. All authors contributed to the article and approved the submitted version.

## Conflict of Interest

The authors declare that the research was conducted in the absence of any commercial or financial relationships that could be construed as a potential conflict of interest.
